# Prediction of Nocturnal Foaling Using Ventral Tail Base Surface Temperature Recorded by a Wearable Device Attached to the Mare’s Tail

**DOI:** 10.3390/ani16020199

**Published:** 2026-01-09

**Authors:** Takahiro Aoki, Guilherme Violin, Tsumugi Jikihara, Makoto Shibata, Shogo Higaki, Tomomi Ozawa, Eri Furukawa, Koji Yoshioka

**Affiliations:** 1 Department of Veterinary Medicine, Obihiro University of Agriculture and Veterinary Medicine, Obihiro 080-8555, Hokkaido, Japan; 2 Athena Integrative Veterinary Care, Obihiro 080-0013, Hokkaido, Japan; 3 National Institute of Animal Health, National Agriculture and Food Research Organization, Tsukuba 305-0856, Ibaraki, Japan; 4 Laboratory of Theriogenology, School of Veterinary Medicine, Azabu University, Sagamihara 252-5201, Kanagawa, Japan

**Keywords:** mare, wearable sensor, ventral tail base surface temperature, foaling, prediction, precision livestock farming

## Abstract

It is known that a mare’s body temperature drops before parturition, but no research has yet applied this thermal change to the prediction of foaling. In this study, the ventral tail base surface temperature (VTB-ST) was recorded by a tail-attached device equipped with a thermistor in pregnant mares kept in an outdoor paddock all day. The objective of the present study was to make an algorithm for predicting nocturnal foaling (18:00 to 6:00) and to verify the accuracy of the algorithm. Prediction of nocturnal foaling was performed at 15:00 every day. The foaling prediction model was validated using 147 days of data recorded from 22 mares. The sensitivity of the foaling prediction model proposed in this study was 68.2 to 81.8% and the precision was 51.4 to 62.5%. To our knowledge, the present study is the first one to establish an algorithm for predicting nocturnal foaling at a specific time interval using VTB-ST. Further study will be necessary to improve the foaling prediction model, as the accuracy of the algorithm proposed in this study was considered to be insufficient for practical use in stud farms.

## Simple Summary

It is desirable for a horse manager to attend to the mare at the time of foaling in order to assist in fetal delivery and prevent complications. However, the gestation length of horses is highly variable, and most mares give birth during the night. These characteristics of equine parturition make it difficult to attend to the foaling mare. It is known that a mare’s body temperature drops before parturition, but no research has applied this thermal change to the prediction of foaling yet. In this study, the ventral tail base surface temperature was recorded by a tail-attached device equipped with a thermistor in pregnant mares kept in an outdoor paddock all day. The objective of the present study was to make an algorithm for predicting nocturnal foaling (18:00 to 6:00) and to verify its accuracy. The foaling prediction model was validated using 147 days of data recorded from 22 mares. When the threshold was set at −0.2 to −0.3 °C, sensitivity was 68.2 to 81.8% and precision was 51.4 to 62.5%. Although the accuracy might be insufficient for clinical application, to our knowledge, this present study is the first to propose an algorithm for predicting nocturnal foaling at a specific time using ventral tail base surface temperature.

## 1. Introduction

The incidence of dystocia in horses is approximately 5 to 10% [1,2,3], and a prolonged second stage of labor caused by dystocia has been reported to increase fetal and neonatal mortality [4]. According to the foaling records (183 births of Japanese draft horses recorded on three stud farms from 2009 to 2013, unpublished data) examined by the authors, the incidence of stillbirth or neonatal deaths was significantly higher in births with no attendance (16.0%, *n* = 4/25) than in births attended by horse managers (3.8%, *n* = 6/158). Therefore, it is desirable for a horse manager to attend to the mare at the time of foaling in order to assist fetal delivery and prevent complications.

The gestation length of horses varies by breed and is more variable than that of other farm animals [5,6,7]. Furthermore, most horses give birth during the night [8,9]. This variability necessitates prolonged human monitoring, which is labor-intensive and costly. Many studies have been performed to predict the date of foaling. The composition of pre-partum milk (udder secretion) dramatically changes as parturition approaches, and based on these changes, pre-partum milk tests as a way to predict the foaling date have been put into practical use [10,11,12]. However, it is often difficult to collect samples from nulliparous or nervous mares, and there are concerns that inexperienced handlers may be at risk of injury, such as being kicked by the mares. Therefore, it is necessary to develop a non-invasive, or even remotely accessible, method for predicting parturition in horses.

The commercial demand for precise animal management technologies incorporating AI (artificial intelligence), ICT (information and communication technology) and IoT (internet of things) is growing in order to address the global shortage of livestock workers. These technologies are useful for monitoring health status and managing reproduction of livestock animals, and are currently available for some animal species in many countries. In horses, wearable devices and image analysis techniques have been studied and developed to detect the onset of labor. Researchers have identified characteristic behaviors associated with the onset of labor, such as lying down and tail-raising behaviors, and were able to successfully detect it through them [13,14]. Nabenishi et al. reported that the onset of labor could be accurately detected by assessing activity intensity and change in body surface temperature using a digital camera system comprising a thermal imaging camera and a visible camera [15]. However, there have been no reports on the successful prediction of labor before the appearance of behavioral signs.

It has been reported that body temperature drops before parturition in cows, sheep, and dogs [16,17,18,19,20], and devices for monitoring body temperature have been developed to predict parturition. In horses, a drop in body temperature has been recognized to occur immediately before parturition [21,22]. A recent study [23] using an implanted microchip to measure body temperature every two hours reported that body temperature drops on the day of parturition, and that evaluating body temperature at 12 h before parturition can predict parturition with relatively high accuracy. However, this method has a potential problem for clinical application, as it is difficult to predict parturition at a fixed specific time. Moreover, differences in horse care management such as all-day grazing or not (stall rest during night) among the studies could have made it difficult to interpret changes in body temperature before parturition. A recent review article explained that management practices impact time-activity budgets such as feeding, resting, standing and locomotion in equids [24], and these behavioral differences could possibly affect biological parameters. Koyama et al. reported that the calving time in dairy cows can be predicted within 24 h in advance by monitoring ventral tail base surface temperature (VTB-ST) using a wearable wireless sensor [17]. In our study, these systems and concepts reported in dairy cows were applied for the prediction of equine parturition.

We hypothesized that VTB-ST, processed as day-to-day differences over a 6 h window (9:00 to 15:00), would decrease on the day of parturition and could be used to predict nocturnal foaling (18:00 to 6:00) with acceptable sensitivity and precision. The objective of the present study was to make an algorithm for predicting nocturnal foaling, as well as to verify the accuracy of the algorithm.

## 2. Materials and Methods

### 2.1. Animals

**Figure 1:**
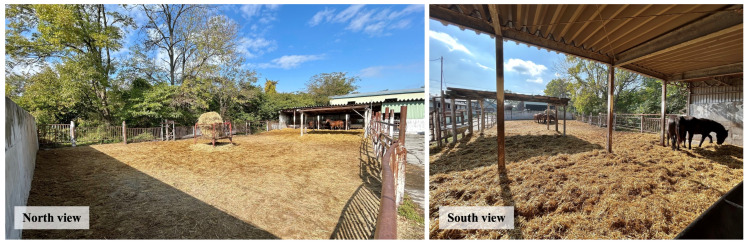
**An outdoor paddock on the farm where the study was conducted.** The paddock (13 × 35 m) had two roofs (4.2 × 13 m, 5.5 × 5.8 m), and straw was laid as bedding material under the roof, which was replaced regularly. All mares used in the study gave birth under these roofs.

This present study was conducted on a private farm in Obihiro city, Hokkaido prefecture, Japan, in 2021 and 2023–2025. Pregnant heavy draft mares (cross-breed mares between Percheron, Belgian and Breton) that were kept in this farm were used in this study. From a few weeks before their due date, pregnant mares were housed in groups of 3–5 mares in a partially covered outdoor paddock (13 × 35 m, Figure 1) 24 h a day.

The mares were fed a concentrate diet (oats 2–3 kg/day, bran 1 kg/day) in the morning (6:00) and evening (17:00), with free access to dry hay, water and mineral blocks (Koen^®^ E250 TZ, Nippon Zenyaku Kogyo Co., Fukushima, Japan). A horse manager regularly watched the mares during the day, and a camera surveillance system was used for monitoring at night. Delivery was confirmed by recorded video and/or human monitoring, and the time of foaling was defined in this study as the time when the fetus was delivered. The dam and foal were moved to a private stall within an hour of giving birth to avoid interference from other mares.

All protocols and procedures were approved by the Animal Care and Use Committee, Obihiro University of Agriculture and Veterinary Medicine (approval no. 20–220, 21–25, 22-48, 23-46, 24-53 and 25-088).

### 2.2. Tail-Attached Device

**Figure 2:**
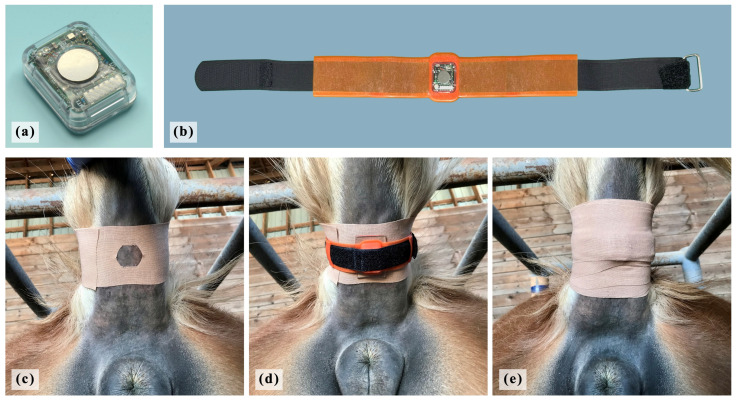
**The sensor, other accessories, and attachment method.** (**a**) The dimensions of the sensors were 26.0 × 21.0 × 9.7 mm, and they weighed 5.8 g with a battery inserted. (**b**) A custom-made silicone belt and hook-and-loop fastener. (**c**) An elastic tape with a window was wrapped around the tail before the silicon belt was attached to protect the skin. (**d**) The tail sensor was attached using a custom-made silicone belt and hook-and-loop fastener, so that the part of the sensor that measured temperature was in close contact with the ventral side of the tail base. (**e**) The sensor was secured in place with medical elastic tape.

The sensor used was a tail sensor developed in previous research [25,26]. The dimensions of the sensors were 26.0 × 21.0 × 9.7 mm, and they weighed 5.8 g with a battery inserted (Figure 2a). The device comprises a thermistor (503ET-3H; SEMITEC, Tokyo, Japan) and wirelessly transmits the data on VTB-ST (in the range of 20 to 45 °C, with 0.05 °C resolution and ±0.3 °C accuracy). The tail sensor was attached using a custom-made silicone belt and hook-and-loop fastener (Figure 2b), so that the part of the sensor that measured temperature was in close contact with the ventral side of the tail base (Figure 2d). Then, the sensor was secured in place with medical elastic tape (Figure 2e). In 2021 and 2023, wounds in the tail’s skin were observed in some mares after the experiment because the sensor and belt were placed directly on the tail. In 2024 and 2025, to remedy this issue, an elastic tape was wrapped around the tail to protect its skin before the silicon belt was attached. This elastic tape had a window on the tail’s ventral skin side to ensure that the thermistor adhered to the skin (Figure 2c).

Although the sensors were attached to the mares from one to two weeks before the due date, they could be attached earlier or later, depending on the degree of mammary gland development. The sensors were removed a few days after delivery. The average gestational age at enrollment was 320 days (302–331 days). The sensor belt and fixing tape were replaced every one to two weeks. The data obtained from the sensor was automatically transmitted to the receiver at 3 min intervals and stored on a cloud server via 3G/LTE. The receiver was placed approximately 2 m high on the wall of a walkway next to the outdoor paddock.

Devices were attached to a total of 57 mares and data were collected, but only data obtained from mares that gave birth at night (18:00 to 6:00) were used in this study. Mares with daytime foaling (*n* = 4), dystocia (*n* = 1), illness (*n* = 2), injury (*n* = 1), and without data for the seven consecutive days before parturition (*n* = 27) were excluded from the calculation. Data on VTB-ST recorded from 22 mares were used in this study. The average age and parity of the 22 mares were 7.8 (4–14) and 2.3 (0–8), respectively. These mares gave birth between February and May, and the mean temperature on the day of birth was 4.8 °C (−6.5 to 15.9 °C). The background information of the mares used in this study is shown in Table S1.

### 2.3. VTB-ST During the Last 5 Days of Pregnancy

In the area where the study was performed, prepartum mares are generally kept outdoors in paddocks or in pastures during the day, and monitored in indoor stalls during the night. Considering future clinical application, the foaling prediction model was proposed using data from the daytime (9:00 to 15:00) before the mares were moved to indoor stalls. The mares used in this study were kept in an outdoor paddock all day long.

**Figure 3:**
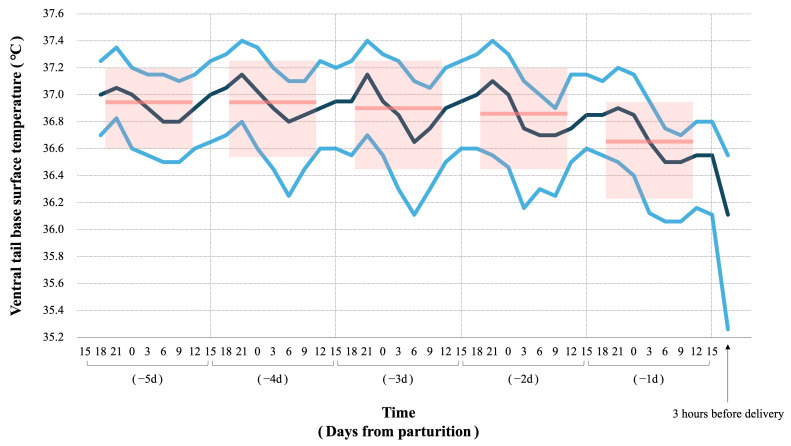
**Changes in VTB-ST every 3 h.** VTB-ST was obtained from 22 mares that gave birth at night (18:00 to 6:00). Data is shown from 5 days before parturition until 15:00 on the day of parturition, as well as data from 3 h before parturition. The numbers on the horizontal axis indicate the actual time of day, except for the data from 3 h before parturition, which is relative to delivery time. The polylines show the 25 percentile (sky blue), median (dark blue), and 75 percentile (sky blue) of every 3 h. The pink bars show the median for every 24 h, and the pink boxes show the range from the 25 to 75 percentiles.

To evaluate daily changes in VTB-ST during the last 5 days of pregnancy, raw VTB-ST values were grouped into 24 h periods starting at 15:00, and descriptive statistics (median, 25th and 75th percentiles) were calculated for each period. Although the data after 15:00 on the day of parturition were excluded from the calculation, the data from three hours before parturition are shown in a graph in Section 3 (Figure 3).

### 2.4. Data Preprocessing of VTB-ST

In order to eliminate errors and to cumulatively analyze the daily differences, the raw data of VTB-ST was processed according to the following steps: extraction of the highest hourly value for each mare; calculation of the difference from the previous day (STd1) and two days before (STd2) for the same time of day; calculation of the average value of STd1 and STd2 over the last 6 h (STd-6h).

The rationale for these preprocessing procedures was based on the pilot study conducted prior to this one. The study found that the horse’s VTB-ST could show errors when the device was temporarily cooled by the environment’s air due to behaviors such as lying down or tail raising. In order to minimize these errors, the maximum value for each hour was used in this study. The pilot study also suggested that the accuracy of the foaling prediction model was relatively lower when using only the difference in VTB-ST from the previous day. Therefore, the differences in VTB-ST from the previous day and two days before were used for the model in this study. The data during daytime (9:00 to 15:00) is less affected by differences in husbandry management between stud farms, and therefore was selected for the model in this study.

### 2.5. Foaling Prediction Model

STd-6h was evaluated at 15:00 every day, and a positive alert was defined as a value below multiple test thresholds (from −0.05 to −0.55, in increments of 0.05 °C). This threshold was set based on a decrease of 0.2 to 0.3 °C in the median value of −1d VTB-ST data when compared to the data from the previous days (as shown in Section 3). The accuracy of predicting the parturition on that night (18:00–6:00) was examined using parameters such as true positive (TP), false positive (FP), false negative (FN), sensitivity and precision. TP was the number of mares that gave birth during the night following positive alert at 15:00. FP was the number of mares that did not give birth during the night following positive alert. FN was the number of mares that gave birth during the night with no positive alert. Sensitivity and precision were calculated according to the following equations:

sensitivity = TP/(TP+FN) ×100; precision = TP/(TP+FP) ×100

Due to the characteristics of this study, the number of true negatives is much larger than the number of true positives. Furthermore, changing the number of days used for validation significantly changes the values of true negative and specificity. In order to properly validate the accuracy of the foaling prediction model, the number of days used for the calculation of STd-6h was set to a maximum of 7 days before parturition. (average number of days to determine STd-6h: 6.7 days, range: 5–7 days).

## 3. Results

**Figure 4:**
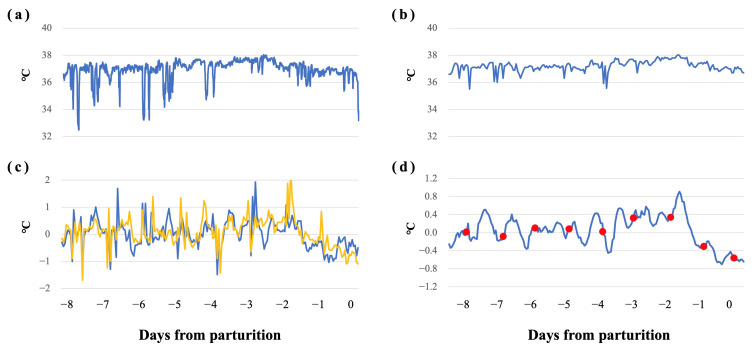
**An example of the smoothing process of VTB-ST**. (**a**) Raw VTB-ST data. (**b**) Maximum value for each hour. (**c**) Difference from the previous day (STd1, blue) and from two days before (STd2, yellow) for the same time. (**d**) Average value of STd1 and STd2 over the last 6 h (STd-6h). The transition can be observed when the data are evaluated at specific time points (red marker).

**Figure 5:**
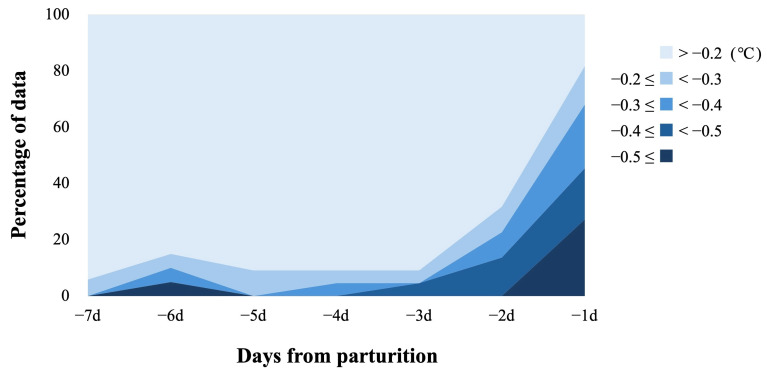
**The distribution of STd-6h calculated at 15:00 in the 7 days preceding foaling.** The mares were classified according to the STd-6h value calculated at 15:00 on each day. The number of mares was 22 for −1d to −5d, 20 for −6d, and 17 for −7d.

The raw data of VTB-ST recorded by wearable devices attached to the mares’ tails are shown in Table S2. The changes in VTB-ST every 3 h are shown in Figure 3. A clear circadian rhythm for VTB-ST was observed. During the 5 days before parturition, the median values of VTB-ST every 3 h were at their highest (36.9 to 37.2 °C) between 18:00 and 21:00 and at their lowest (36.5 to 36.7 °C) between 3:00 and 6:00. The median values of the raw VTB-ST data for each 24 h period were 36.95 °C, 36.95 °C, 36.9 °C, 36.85 °C, and 36.65 °C for −5d, −4d, −3d, −2d, and −1d, respectively. An example of the preprocessing of VTB-ST is shown in Figure 4. The calculated values after preprocessing are shown in Table S3. The distribution of STd-6h calculated at 15:00 is shown in Figure 5. The percentage of mares with STd-6h ≤ −0.2 °C was 5.9% for −7d, 15% for −6d, 9.1% for −5d to −3d, 31.8% for −2d and 81.8% for −1d, respectively.

The foaling prediction model was validated using 147 days of data recorded from 22 mares. Foaling occurred on 22 days of 147 days. TP and FN were assessed based on 22 foaling days. FP was assessed based on 125 non-foaling days. The validation results for foaling prediction accuracy are shown in Table 1. Considering the balance between sensitivity and precision, a threshold of −0.2 to −0.3 °C seemed the most promising. At this threshold, sensitivity ranged from 68.2 to 81.8% and precision from 51.4 to 62.5%. At the −0.2 °C threshold, the model triggers an average of 1.6 alerts per mare over 7 days, of which approximately half correspond to actual foalings.

## 4. Discussion

Many attempts have been made to detect the onset of labor in horses using wearable sensors. However, to our knowledge, this present study was the first to establish an algorithm for predicting nocturnal foaling at a specific time interval using VTB-ST. This study revealed that continuous evaluation of VTB-ST can predict nocturnal foaling with moderate accuracy.

In our pilot study, the authors confirmed that VTB-ST exhibits a clear circadian rhythm. Therefore, it was necessary to consider day-to-day changes at the same time point when proposing an algorithm for foaling prediction. The period of 9:00 to 15:00 was selected for evaluation because it was less susceptible to the effects of husbandry management in this study. This time interval coincides with the rising period in VTB-ST within the circadian rhythm. Compared to the surface temperature of other parts of the body, such as the neck or chest, VTB-ST might be less affected by environmental temperature. The VTB-ST obtained in this study indicates values closer to core body temperature because the sensor was sandwiched between the skin of the ventral tail base and the outside of the anus. In our pilot study, a strong positive correlation was found between VTB-ST and rectal temperature (*r* = 0.86, *p* < 0.01, *n* = 89, unpublished data). Meanwhile, in a previous study with dairy cows using the same tail sensor [17], it was confirmed that the alterations observed in pre-calving VTB-ST change in pattern between the warm season (average air temperature of 10 to 20 °C) and the cool season (<10 °C). Moreover, the accuracy of the calving prediction model was also affected by the seasons. The mares used in this study were bred for the production of draft racing horses (Ban’ei in Obihiro city, Japan), so foaling dates were concentrated between February and May, the first half of the equine breeding season. This background makes it difficult to collect samples during the hot season. The average temperature on foaling days in this study was 4.8 °C (−6.5 to 15.9 °C). It will be necessary to collect data on the same horse breed in warm regions and re-examine the influence of environmental factors on the change in pattern of the pre-partum VTB-ST alterations for future clinical application.

The algorithm developed in this study for predicting parturition in horses is based on the gradual drop in body temperature that occurs from about 24 to 48 h before parturition, rather than the sudden drop in body temperature that occurs just before parturition. Although the mechanism behind the thermal drop that occurs before parturition is not yet fully understood, the involvement of progesterone has been suggested in some animal species. Cagnacci et al. [27] found that estradiol has a hypothermic effect in post-menopausal women (*n* = 6), and this influence may be mediated through endogenous opioids at the hypothalamic level, but progesterone has a hyperthermic effect on body temperature that seems to be independent of endogenous opioids. Lammoglia et al. [28] examined peripartum thermal changes using electronic temperature monitors surgically placed under the left flank’s obliquus abdominis internus muscle in 7 multiparous crossbred beef cows. They reported that body temperature significantly decreased from 48 h to 8 h before parturition, which coincided with a decrease in progesterone and an increase in PGFM. Veronesi et al. [29] measured body temperature twice daily and assessed blood hormone concentrations once daily in 7 periparturient bitches. In this study, no significant decrease in body temperature occurred before parturition, but there was a significant increase in blood 15-ketodihydro-PGF2a and a significant decrease in progesterone 24 h before parturition. Geiser et al. [20] inserted a temperature logger into the vaginal cavity of 16 pregnant bitches at the end of pregnancy and continuously evaluated changes in body temperature until the day of parturition. Although they found a decrease in vaginal temperature before parturition, the accuracy of predicting parturition based on vaginal temperature alone was not so high, as individual temperature changes not corresponding with parturition were observed. Katsumata et al. [30] evaluated peripartum changes and interactions between body temperature and circulating progesterone concentration in Killer whales. This study found a positive correlation between core body temperature and blood progesterone levels during the hyperthermia phase observed in early pregnancy. Body temperature subsequently gradually decreased, but no correlation was found between body temperature and blood progesterone levels during this period. Furthermore, a significant decrease in body temperature was observed several days before parturition, but no correlation with progesterone levels was confirmed. This suggests that the decrease in body temperature before parturition may not be explained solely by endocrinological factors. Although equine gestation length can be as long as 11 months [5,6,7], the final stages of fetal maturation are thought to occur rapidly during the 2–3 days before parturition. Maternal total progesterone concentrations increase during the final few weeks of gestation and then decline precipitously during the last few days, or even hours, before delivery [31]. The pre-partum rise in progesterone is associated with the development of the mammary gland and mammary secretions, while the decline is concurrent with an increase in fetal cortisol [31]. The trigger for these changes and the effect of endocrinological changes on body temperature remain unclear, and further research is necessary to clarify the mechanisms behind parturition in mammals.

The algorithm proposed in this study is unlikely to achieve the high accuracy required for clinical breeding management. At a threshold of −0.2, it can predict more than 80% of nocturnal births (sensitivity 81.8%), but on average, there will be one false alarm per mare (precision 51.4%) during the last 7 days of pregnancy. A previous study [14] suggested that prolonged lying posture possibly leads to the cooling of the device by colder environmental air, because lying posture allows an interspace between the ventral tail base skin and the anus. In addition, since this study was conducted using mares kept in an outdoor paddock all day, the sensor might be cooled down by rain or snow. These factors are likely the cause of a certain percentage of false positives and false negatives. It may be difficult to simply compare the accuracy of the foaling prediction method proposed in this study with previously reported methods because the sampling period, sampling time, and analysis method were different. On the other hand, pre-partum milk testing is useful for predicting the date of parturition in mares. Diel de Amorim et al. examined milk samples collected during the last four days before foaling in the evening (from 18:00 to 21:00) using several methods to verify their accuracy in predicting the parturition. That study found that calcium measured by using the titration-based test method (sensitivity 87.2%, specificity 66.3%), pH obtained using the pH digital meter (sensitivity 65.8%, specificity 71.6%), and pH obtained using the pH indicator paper strip (sensitivity 55.0%, specificity 88.0%) were useful for predicting parturition in Standardbred mares [12]. If tail sensors are to be used clinically in the future, the accuracy of the foaling prediction model must be better than that of the prepartum milk test. Our previous study [14] found that the tail-attached device with a thermistor and tri-axial accelerometer could detect the onset of labor with 100% sensitivity and accuracy by using data on VTB-ST together with lying and tail-raising behavior. Therefore, the combination of the previously reported foaling detection model with the foaling prediction model developed by the present study could contribute to a more efficient foaling management with less labor. Predictive algorithm helps horse breeders to prepare for the nocturnal foalings. Even if the predictive model does not work well for some mares, the delivery detection model will certainly inform the onset of labor early. Nabenishi et al. analyzed camera images of prepartum mares housed in indoor stalls during the night and reported that their locomotion distance increased 2 to 3 h before the onset of labor [15]. That study, however, did not provide behavioral information during the daytime because the mares were moved to a pasture between 7:00 and 15:00. Analyzing the activity data obtained from tail sensors using AI tools such as machine learning and combining them with thermal algorithms could possibly improve the current foaling prediction model.

One of the limitations of this study is the lack of external validation. The dataset used in this study was used to develop the algorithm, and the same dataset was used to verify its predictive accuracy. Therefore, there is a potential for overfitting, a possibility that this model is only applicable to the specific farm used in this study. For future clinical application, external validation is essential, and the authors have plan to collect data from other farms to verify the quality of the algorithm. Another limitation is the small size of the dataset. This study was based on a limited number of Japanese heavy draft mares (*n* = 22) with nocturnal foaling at a farm. To generalize the algorithm for foaling prediction, it will be necessary to collect and verify more data including daytime foaling in a variety of farms.

## 5. Conclusions

The foaling prediction model proposed in this study was found to be able to predict around 80% of nocturnal foalings by calculating the VTB-ST from 9:00 to 15:00 on the day of parturition. This algorithm may help reduce night-time monitoring load, but due to the moderate precision and incompleteness of the model, it cannot yet replace human supervision. In the future, it will be necessary to re-examine its performance using mares kept in various environments on multiple farms.

## Figures and Tables

**Table 1:** The foaling prediction model was validated using 147 days of data recorded from 22 mares. STd-6h was calculated at 15:00 each day, and a result below the threshold was defined as positive. The sensitivity and precision were calculated according to the following equations: sensitivity = TP/(TP + FN) × 100; precision = TP/(TP + FP) × 100.

Threshold (°C)	^1^ TP (*n*)	^2^ FP (*n*)	^3^ FN (*n*)	Sensitivity (%)	Precision (%)
−0.05	20	47	2	90.9	29.9
−0.1	20	38	2	90.9	34.5
−0.15	19	28	3	86.4	40.4
−0.2	18	17	4	81.8	51.4
−0.25	16	15	6	72.7	51.6
−0.3	15	9	7	68.2	62.5
−0.35	11	6	11	50.0	64.7
−0.4	10	5	12	45.5	66.7
−0.45	8	4	14	36.4	66.7
−0.5	6	1	16	27.3	85.7
−0.55	5	1	17	22.7	83.3

^1^ TP = True positives: number of mares that gave birth during the night following positive alert at 15:00; ^2^ FP = False positives: number of mares that did not give birth during the night following positive alert; ^3^ FN = False negatives: number of mares that gave birth during the night with no positive alert.

## Data Availability

All relevant data are within the manuscript and its Supplementary Materials files.

## Supplementary Materials

The following supporting information can be downloaded at https://www.mdpi.com/article/10.3390/ani16020199/s1, Table S1: Background information of the mares used in this study; Table S2: Raw data of VTB-ST recorded by wearable devices attached to the mares’ tails; Table S3: Calculated values after preprocessing.
